# Accuracy and efficiency of detection dogs: a powerful new tool for koala conservation and management

**DOI:** 10.1038/srep08349

**Published:** 2015-02-10

**Authors:** Romane H. Cristescu, Emily Foley, Anna Markula, Gary Jackson, Darryl Jones, Céline Frère

**Affiliations:** 1 GeneCology Research Centre, University of the Sunshine Coast, Maroochydore DC, Queensland, Australia 4558; 2 Environmental Futures Research Institute, Griffith University, Nathan, Queensland, Australia 4111; 3 Logan City Council, 150 Wembley Road, Logan Central, Queensland, Australia 4114; 4 G‌ary Jackson Dog Trainer, 190 McPhail Road, Narangba, Queensland, Australia 4504

## Abstract

Accurate data on presence/absence and spatial distribution for fauna species is key to their conservation. Collecting such data, however, can be time consuming, laborious and costly, in particular for fauna species characterised by low densities, large home ranges, cryptic or elusive behaviour. For such species, including koalas (*Phascolarctos cinereus*), indicators of species presence can be a useful shortcut: faecal pellets (scats), for instance, are widely used. Scat surveys are not without their difficulties and often contain a high false negative rate. We used experimental and field-based trials to investigate the accuracy and efficiency of the first dog specifically trained for koala scats. The detection dog consistently out-performed human-only teams. Off-leash, the dog detection rate was 100%. The dog was also 19 times more efficient than current scat survey methods and 153% more accurate (the dog found koala scats where the human-only team did not). This clearly demonstrates that the use of detection dogs decreases false negatives and survey time, thus allowing for a significant improvement in the quality and quantity of data collection. Given these unequivocal results, we argue that to improve koala conservation, detection dog surveys for koala scats could in the future replace human-only teams.

Accurate data on presence/absence and spatial distribution for fauna species is fundamental to conservation biology^1^. Despite this, acquiring such critical data can often be time consuming, laborious and costly, all of which are exacerbated for fauna species characterised by low densities, large home ranges and cryptic or elusive behaviour^2^,^3^,^4^. For these species, such as pine martens (*Martes martes*), red fox (*Vulpes vulpes*) or koalas (*Phascolarctos cinereus*), indirect methods are often relied upon, the most common being surveys of faecal pellets (scats)^5^,^6^.

Obtaining a false negative, or the inability to detect a species when it is indeed present, is a recurrent concern in ecology and can lead to dramatic consequences, such as errors in the interpretation or predictions of distribution or habitats favoured by species^7^,^8^. As a consequence, caution is advocated in interpreting estimates of occupancy when sample sizes are small and the number of repeated visits is less than seven^9^,^10^. Such a high number of repeated visits can make many surveys logistically unfeasible. Scat surveys, while heavily relied upon, are unfortunately not exempt from false negatives^11^. In particular, detection of scats can be difficult as researchers surveying for scats rely on their eyesight and scats are easily obscured by ground cover. For example, the koala, a cryptic arboreal Australian marsupial often found at low densities, produces small sized scats (1.5 by 0.5 cm) which are easily hidden from view in dense ground vegetation or covered by leaves, are usually difficult to locate, and whose detectability by humans decreases as the complexity of ground cover increases^11^. However, the scat's odour persists and dogs, due to their high olfaction ability, represent a potentially powerful alternative method for scat survey^12^. Ground cover complexity can affect the availability of scent for detection dogs but this does not consistently impact on the ability of detection dogs to detect a target^13^. For instance, the performance of detection dogs to locate bat carcasses does not seem to be affected by ground cover complexity^14^. Thus detection dogs may provide the double advantage of an increased detection rate with a decreased bias.

As a result, detection dogs are increasingly used in conservation surveys, including for scat surveys focusing on carnivores such as black bears (*Ursus americanus*), kit foxes *(Vulpes macrotis mutica)* or bobcats (*Lynx rufus*)^15^,^16^, yet this practice is rarely employed in Australia, and has not previously been applied to the detection of koala scats.

Developing better conservation tools for the protection of the koala is critical given that in 2012 it was classified as a nationally vulnerable species under the Australian Government's *Environment Protection and Biodiversity Conservation Act 1999*. Koala numbers are declining across much of the remaining populations in mainland Australia^17^ due to processes threatening koalas including susceptibility to disease (e.g. Chlamydia), habitat fragmentation, urban development, road-associated deaths and attack by domestic dogs^18^. Despite such alarming trends, we are still lacking comprehensive data regarding the koala's current distribution across its range.

Here, we used experimental and field-based trials to investigate the accuracy (defined as the number of finds divided by number of opportunities^19^) and efficiency (time to find scats^20^) of a detection dog specifically trained for koala scat surveys.

## Results

### Experimental Trial

In 150 trials where scat locations were known by a third party, the detection dog found and indicated the location of koala scats 146 times (97% success rate). All failures to find scats occurred when the detection dog was leashed. The average time to find the scats was 56 sec (SE = 54, Table 1) with a minimum and maximum time of 2 sec and 5 min.

The low number of failures meant that we were unable to analyse whether failures were influenced by the presence of the leash, scat age, number of scats, or the distance from the scats to the transect or along the transect. Instead, we modelled the time taken in each successful trial to find scats. No variable was found to influence time to detect scats (Table 2). Indeed, the model that included only scat positions (along transect and away from transect) was significantly better than any others (ΔAIC > 4). However, time to detect scats was only correlated with the distance of the scats along the transect (p < 0.001) with a coefficient close to zero (β = 0.067 SE = 0.008).

Validations of the model included goodness-of-fit and residual analyses. The goodness-of-fit of the global model of time to detect scats was significantly better than a model with just the intercept (likelihood ratio test, χ^2^ = 24.90, p < 0.001). Residuals plotted against fitted values and each explanatory variable showed no patterns, the histogram of the residuals was normally distributed and the QQ-plot was normal.

### Field trial

**Figure 1:**
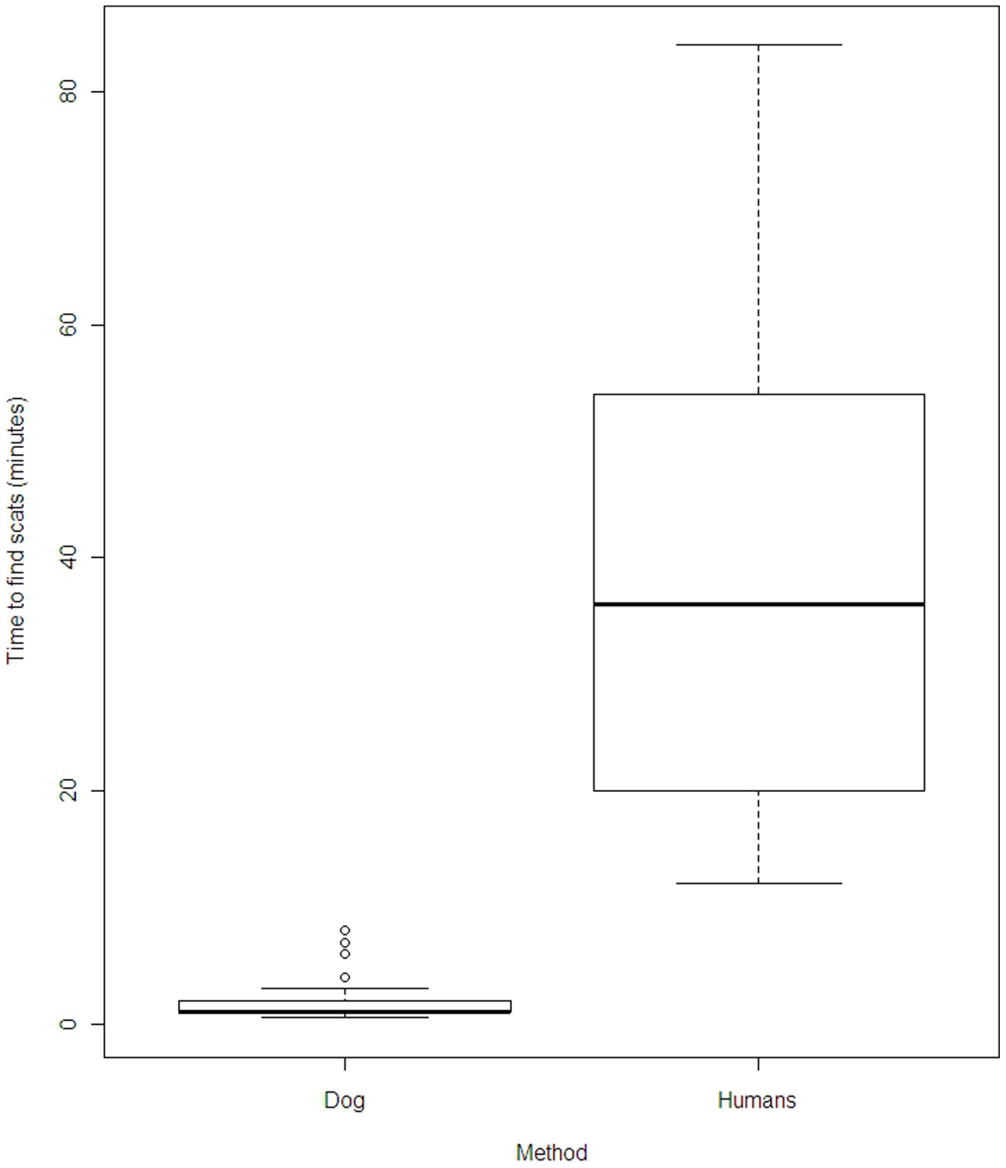
Difference of time (in minutes) between the detection dog method and the human-only method to find scats.

**Figure 2:**
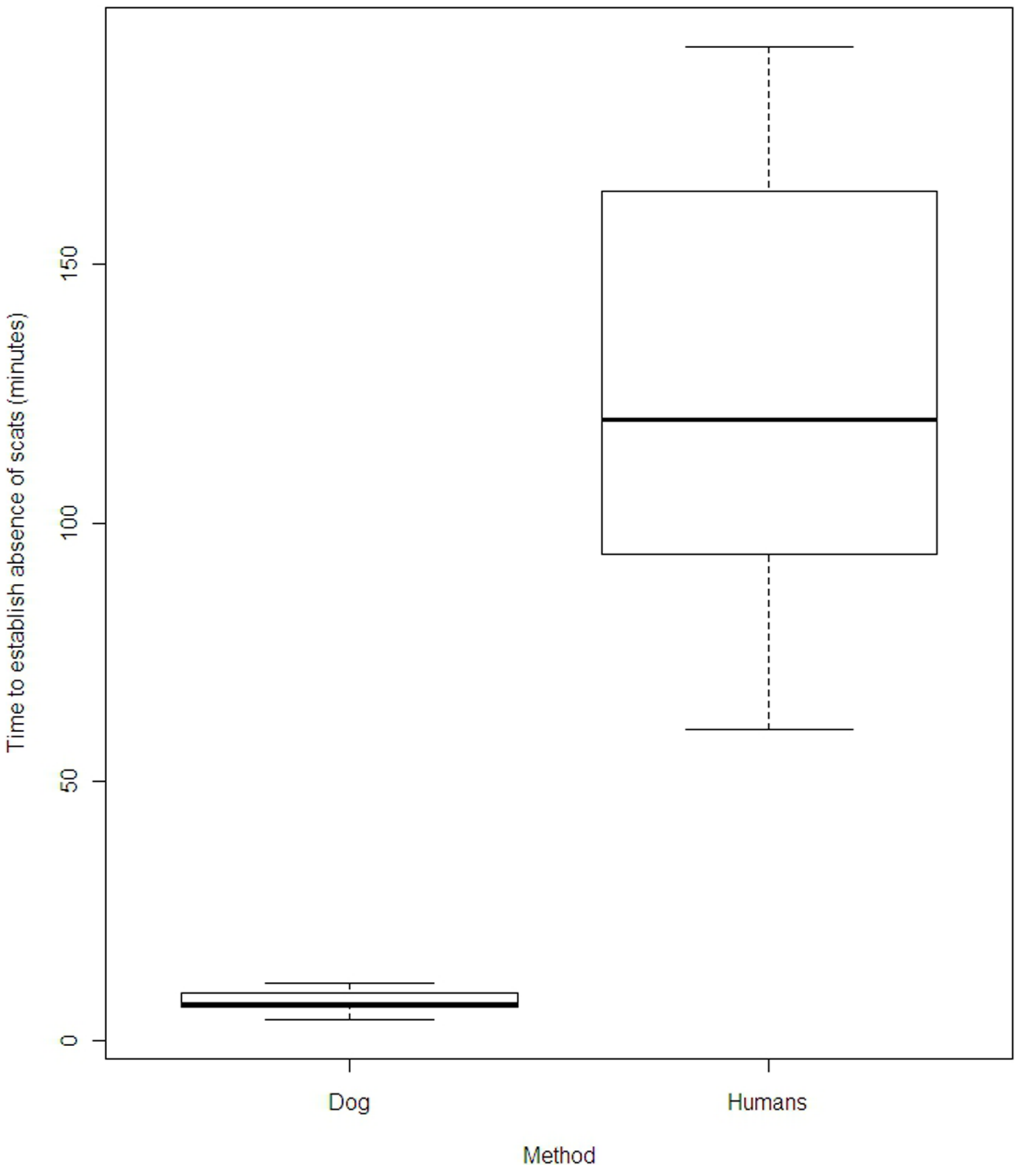
Difference of time (in minutes) between the detection dog method and the human-only method to establish the absence of scats.

Overall, we found that the detection dog team had 24% less false negative results than the human-only team, which translates to a 153% increase in accuracy rate. Indeed, the detection dog team detected koala scats in eight locations where the human-only team did not. In contrast, the human-only team failed to detect scats anywhere the detection dog team was unsuccessful (Table 3). The detection dog team method was also significantly more efficient than the human-only team (Wilcoxon signed-rank test, p = 3.995e-06). Indeed, the search of the detection dog team was on average 19 times faster than the human-only team. In addition, the detection dog team was more efficient whether scats were present (Wilcoxon rank sum test p = 4.903e-08) or absent (Wilcoxon rank sum test p = 4.985e-07). Both methods took longer to establish the absence of scats than to find scats (Table 3, Figures 1 and 2).

## Discussion

Results from the experimental trial of the koala scat detection dog proved that the detection dog team was highly successful at finding koala scats. With the detection dog off-leash, the accuracy reached 100%. Comparing the detection dog results to a previous study on human survey scat detection^11^ revealed that in an experimental setting, the dog was as accurate but much more efficient than a human being (Table 4). In fact the detection dog was on average 350 times quicker. This comparison has some limitations, as the previously mentioned experimental survey^11^ was extremely thorough (up to 49 minutes for a 5 m^2^ plot), whereas consultancy projects would be more limited by working within budget constraints which undoubtedly limits search time. In order to test the performance of the detection dog in a more realistic field survey, we compared its performance to that of a human-only team under a commonly used scat survey method. Not only did we find that the detection dog team was still 19 times more time-efficient, more importantly we found a 24% difference in false negative rates, which means the detection dog team was 153% more accurate than the human-only team. As such, our study shows unequivocally that the detection dog team consistently outperforms human-only teams in efficiency and/or accuracy.

Within the boundaries tested in the control trial, age did not influence the probability of scat detection. Opportunistic observations confirmed that even scats older than in this study, decomposed and weathered (or even burnt scats), can be detected by the dog (R. Cristescu, pers. observation).

The negative influence of the leash on the performance of the dog in the control trial mainly stems from the restriction to the dog movements due to the physical connection to the handler. In particular, when the dog detects a scent, it has to follow the cone of scent to its origin, which the dog may have passed already, i.e. the dog does not travel in a linear search pattern. The leash also can become entangled and waste time or break the search. As a result the leash seemed to decrease the dog's drive. The difficulties of working with a dog on-leash also increase with wind force^21^. However, in the field trial, the detection dog, although frequently on leash, still out-performed the human-only method routinely used for scat survey.

The development of more efficient and accurate tools for the conservation of threatened species is important given that in today's political climate, economics necessarily dictates many conservation decisions^22^. Here we show that a koala survey team which utilises a detection dog will ultimately save time and thus money. We compared the cost of using the detection dog team to the human-only team comprised of four local government employees, for the amount of time each survey method took. Acknowledging that each specific survey would provide different figures, in this case, per location, the detection dog method was more than six times cheaper for the client than the human-only method. For the contractor using a dog, however, the investment in training and maintaining the detection dog also needs to be taken into consideration.

Detection dogs have also been proven to be more cost effective than other survey methods such as camera traps, hair analyses or play-back calls^23^,^24^. The decreased cost in addition to increased accuracy will deliver surveys that can cover larger areas with higher confidence levels than could be undertaken by human-only teams. This undoubtedly would enhance knowledge on koala distribution which would facilitate better-informed management decisions^25^.

The 24% reduction in the rate of false negative results of the detection dog team compared to the human-only team in the field trial would greatly influence the quality of survey data on presence/absence of koalas. This would have a significant impact on the accurate development of koala distribution maps and would therefore bias any downstream analyses such as koala habitat preference and the influence of habitat variables on koala presence. For example, in a simulation paper^7^, Gu and Swihart compared the impact of imperfect detection (i.e., false negative) on habitat variables. They fitted models predicting species distribution based on two habitat covariates and determined the effect of imperfect detection on the estimates of these two covariates. For the highest imperfect detection rate in their models (15% to 20%), the bias on the habitat covariates was of several 100%. Thus we would expect similar or worse trends given that we found that the human-only team had at least 24% false negative results.

Spatial patterns of predicted species distributions are also greatly affected by imperfect detection. Species distribution models notoriously perform badly with imperfect detection^26^. Occupancy models that account for imperfect detection are therefore gaining popularity (see for instance^27^) but they are not always better at predicting species distribution^28^. This is why decreasing false negatives created by imperfect detection remains the most effective way of getting accurate distributions.

We have demonstrated that a detection dog is a powerful method to study koala presence/absence and its use could greatly improve our ability to protect and conserve the koala. This is a preliminary assessment of this new survey method for koalas and clearly shows promising results. However, results of accuracy and efficiency of detection dogs will vary with both the dog and the handler abilities^29^,^30^. This needs to be tested once additional koala scat detection dogs are operational. Importantly, we strongly believe that to ensure that future koala scat detection dogs are to the highest standards (accurate, consistent, no threat to wildlife), national standards and tests must be developed.

Scats also contain a suite of important ecological information such as diet^31^, genetics^32^,^33^, physiological stress and reproductive activity^34^. Although diet information can be extracted from koala scats (see for example^35^), more work remains to be done to ascertain whether koala scats can be used to extract other ecological information such as genetics, physiological stress, reproductive activity and maybe even more importantly, Chlamydia, a disease highly prevalent in koalas^36^. The combination of gathering data on koala distribution and of performing ecological assessments of koala populations based on scat surveys alone would represent a paradigm shift toward non-invasive yet high-data yielding methods in koala research.

## Methods

### Training

Maya, an adult female Border collie cross, was selected in July 2011 and trained by Gary Jackson, a professional dog trainer, to detect and indicate the presence of koala scats. Maya was selected based on her intense attachment to tennis balls (the reward) and a high level of personal motivation (also referred to as the dog's “drive”^19^). Maya was trained to associate koala scat scent to her reward. Koala scats were hidden along a scent line, and as soon as Maya smelled koala scats, a reward was provided. This conditioned her to search intensively for koala scat scent. Next, Maya was trained to indicate when she found a koala scat scent using a specific trained alert: dropping. This allowed the handler to discern when the scent had been located in a relatively non-invasive way (i.e. barking was avoided as that could increase wildlife stress during field work). Once the dog was deemed efficient in a controlled environment, training continued outdoors with the handler (Dr Romane Cristescu). When Maya performed consistently well in different outdoor environments and showed no interest to the surroundings wildlife and other potential distractions, the trials of this study begun.

The trials were performed in accordance with relevant guidelines and regulations and approved by the Community Access Animal Ethic Committee CA 2012/05/610 and the DEHP Scientific Purpose Permits WITK1167712 and WISP11677512.

### Experimental Trial

Experimental trials were conducted on Minjerribah (North Stradbroke Island), Queensland, Australia in eucalyptus bushland between May-August 2012. These trials consisted of the dog and handler team searching for scats, the locations of which were known only to a third party. A number of scats (varying from 1 to 5) were deposited by the third party, independent of the handler or the dog knowledge, along a 25 m transect. The position where scats were deposited was randomly chosen (randomly generated number between 0 and 25 m) and the distance from the transect search line varied between 0 m (scats on the transect line), 1 m or 2 m perpendicular to the transect line. Scats were collected fresh (i.e. still covered in mucus) from wild koalas on North Stradbroke Island so that the age of the scats was known. This allowed the alternate use of fresh and old scats in order to test for age effect. Scat age varied from 0 to 91 days old. Scats were kept in a dry place between trials.

The handler walked the entire transect with the detection dog being on or off leash. While off-leash, the dog was redirected when she was moving away from the established transect. The outcome of the search (success or failure) and the time to success were recorded.

Models were constructed, compared and validated in R 2.12.0 (R Foundation for Statistical Computing, Vienna, 2010). Prior to the inclusion of any variables in the models, we tested for collinearity using variance inflation factors (VIF) and no variable had to be removed (all VIF < 5). We had planned to model success/failure to detect scats, but were unable to do so given the low failure rate (see results). The models with the time to detect scats were analysed with a linear regression. Time was log transformed. We created a priori models^37^ with only the use of the leash, only scat characteristics (age in days and number of scats) or only scat positions (along transect and away from transect) and compared these to the global model (see Table 2). The occasion (specific date when different trials were conducted) was used as a random effect. We estimated the global goodness-of-fit between the global model and the null model with a likelihood ratio test^38^. To rank the models, we used Akaike Information Criterion^39^. Model validation included plotting the Pearson residuals of the best model against the fitted values and against each explanatory variable (included or not included in the model), the histogram of the Pearson residuals and the QQ Plot of sample against theoretical quantiles.

### Field Trial

The field trial was designed to compare the scat surveying performance of the detection dog team to a human-only team surveying scats by a consensus method routinely used in koala studies. The location of scats was unknown to both teams, as is normal in a scat survey.

The trial occurred in a total of 33 locations in nine parks across the Logan City Council local government area in Queensland, Australia. At each park, four locations were assessed, 250 m apart along a fire trail, bike track or path and 50 m perpendicular to the trail into the bush – this 1 km strip was often the length of the park. At each location up to 30 trees were searched. The first tree was chosen as the closest to the point reached in the field, with the subsequent 29 trees closest to the central tree being searched. Four people performed the searches in the human-only team, so their time was added to calculate “time to find scats” or “time to establish the absence of scats”. Due to terrain constraints, three parks had only three locations assessed. The two survey methods (detection dog versus human-only) were trialled on the same locations and the same trees in a four-week period.

The human-only team surveyed koala scats based on the Koala Rapid Assessment Method^40^ which is used widely in South East Queensland by the State agencies to map koala habitat (see for instance “South East Queensland Koala Habitat Assessment and Mapping Project” by the Department of Environment and Resource Management of Queensland, 2009). The search was focused on the base of up to 30 trees per location, and was stopped when koala scats were found or the 30 trees had been assessed, with no time limit on the search^11^.

The detection dog team then repeated the search of the 30 same trees. To ensure that the dog was searching the same trees as the human-only team, the dog was leashed, except when sites had thick ground cover that made searching while restrained difficult. As with the Koala Rapid Assessment Method, searching stopped when koala scats were found or when the 30 trees had been searched thoroughly.

The outcome (presence or absence of scats) of the search and time of search (for 28 locations) were recorded for both methods at each location.

We compared the efficiency of both scat survey methods by a non-parametric paired test (Wilcoxon signed-rank test). We also compared the efficiency of both methods when scats were found and when no scat was found with non-parametric Wilcoxon rank sum tests.

## Author Contributions

R.H.A., A.M. and E.F. designed experiments, conducted field work and analysed data. G.J. and R.H.A. trained the detection dog. R.H.A. wrote the manuscript. C.F. analysed the data. D.J. and A.M. supervised E.F. All reviewed the manuscript.

## Figures and Tables

**Table 1:** Variables recorded in the experimental trials (N = 150) where the detection dog searched for koala scats in known locations along transects. The variables were scat number and age (days), the distance of scats from the transect (meters), the position of the scats along the transect (meters), whether the dog was leashed and the time taken by the detection dog to find koala scats

Variables	Range	Average	Standard deviation
Distance from transect (meters)	0–2	1	0.8
Scat age (day)	1–91	27	25.8
Scat number	1–5	3	1.8
Position along transect	0–25	13	6.9
Time (sec)	2–313	56	53.2
Leash	0 or 1	NA	NA

**Table 2:** Models used to determine the influences of scat number and age (days), the distance of scats from the transect (meters), the position of the scats along the transect (meters) and whether the dog was leashed on the time taken by the detection dog to find koala scats

Model	log likelihood	Number of parameters	AIC	ΔQAIC	QAIC weight	Evidence ratio
time ~ distance + position |Occasion	−172.8	5	355.6	0.0	1.0	1.00
time ~ leash + distance + age + number + position |Occasion	−179.4	8	374.8	19.1	0.0	1.43E + 04
time ~ 1 |Occasion	−191.8	3	389.7	34.0	0.0	2.47E + 07
time ~ leash |Occasion	−192.0	4	391.9	36.3	0.0	7.53E + 07
time ~ age + number |Occasion	−197.2	5	404.4	48.7	0.0	3.83E + 10

**Table 3:** Comparison of the detection dog method to the human-only method in a field trial of N = 33 locations (SD = standard deviation), both methods focused on searching the same 30 trees at each location for koala scats

	Locations with koala scats	Average time to search 30 trees in minutes (SD)	Total time to search all locations in hours	Average search time to a find in minutes (SD)	Average search time for absence of scats in minutes (SD)
Detection dog	23	4.2 (3.5)	2.0	2.0 (1.9)	7.6 (1.9)
Human only	15	80.9 (57.3)	37.7	39.2 (22.8)	129.1 (41.8)

**Table 4:** Comparison of accuracy (defined as the number of finds divided by number of opportunities^19^) and efficiency (time to find scats^20^) of a koala ecologist^11^ and a detection dog performing koala scat surveys

	Trained dog	Trained ecologist
Number of trials	150	30
Accuracy	0.97	0.93
Average time in min per 100 m^2^ (standard deviation)	1 (1)	356 (290)
Maximum time in min per 100 m^2^	5.2	972
